# Association between preoperative neutrophil-to-lymphocyte ratio and the survival outcomes of esophageal cancer patients underwent esophagectomy: a systematic review and meta-analysis

**DOI:** 10.3389/fonc.2024.1404711

**Published:** 2024-08-19

**Authors:** Xun Wu, SiJie Liu, FengWei Li, YingTai Chen

**Affiliations:** Department of Thoracic Surgery, Beijing Aerospace General Hospital, Beijing, China

**Keywords:** neutrophil, lymphocyte, NLR, esophageal cancer, esophagectomy, meta-analysis

## Abstract

**Objectives:**

The purpose of this study was to assess the association between preoperative neutrophil-to-lymphocyte ratio (NLR) and the survival outcomes of esophageal cancer patients who underwent esophagectomy, the latest and comprehensive systematic review performed.

**Methods:**

Related literature retrieved from PubMed, Web of Science, Embase, and Cochrane before January 2024, according to the inclusion criteria. Outcomes measured were overall survival (OS), disease-free survival (DFS), relapse-free survival (RFS), and cancer-specific survival (CSS).

**Results:**

Eighteen studies with 6,119 esophageal cancer patients were retained for analysis. Meta-analysis demonstrated that OS (HR: 1.47; 95% CI: 1.29, 1.67; *P* < 0.00001), DFS (HR: 1.62; 95% CI: 1.29, 2.05; *P* < 0.0001), and CSS (HR: 1.62; 95% CI: 1.29, 2.05; *P* < 0.0001) were significantly shorter in the high NLR group compared with the low NLR group. In addition, meta-analysis revealed a similar RFS (HR: 1.47; 95% CI: 0.92, 2.35; *P* = 0.10) among the two groups. Subgroup analysis of OS and DFS based on mean/median age, NLR cutoff, and region found that all subgroups remained significant difference between two groups.

**Conclusion:**

Among esophageal cancer patients who underwent esophagectomy, preoperative NLR can be used as prognostic factor independently. High-preoperative NLR is associated with poor prognosis. More large-scale, multicenter prospective clinical studies are needed to further validate the relationship between preoperative NLR and prognosis of esophageal cancer.

## Introduction

As an increasingly global health problem, esophageal cancer significantly affects human life and survival. Based on the latest data, esophageal cancer ranks the sixth among cancer-related causes of death across the world (1), resulting in over 500,000 cancer deaths annually. With a 60-fold difference between high- and low-incidence regions, esophageal cancer demonstrates a significantly varied distribution, contributing to 5.3% of all cancer-related deaths globally (2, 3). Surgery is currently the preferred treatment for patients with esophageal cancer, often supplemented by adjuvant chemoradiotherapy or immune and targeted therapy (4). However, the overall prognosis and life quality of esophageal cancer patients are poor, with a long-term survival rate of less than 25%, largely because symptoms or signs typically appear when the disease has already advanced to a late stage (5, 6). Esophageal cancer patients experiencing poor prognosis primarily due to the hidden symptoms experienced by early stage patients, the absence of clear tumor specific markers in clinical practice, and the lack of specific and sensitive screening programs (7, 8). Therefore, the prognosis of esophageal cancer patients is unfavorable, and there is an urgent demand for specific indicators capable of predicting tumor occurrence and prognosis (9).

Since Virchow first proposed the potential correlation between inflammation and tumors in the 19th century (10), more and more epidemiological data and molecular biology laboratory tests have revealed the close relationship between the tumor microenvironment and tumor development. C-reactive protein, neutrophils, and lymphocytes within the tumor microenvironment can induce cellular oxidative stress, DNA mutations, proliferation, invasion, metastasis, and other associated reactions. This process ultimately fosters tumor initiation and progression, thereby influencing patient prognosis (11–14). Emerging studies on inflammatory complex indexes, such as the neutrophil-to-lymphocyte ratio (NLR), are shedding light on their correlation with tumor occurrence, proliferation, invasion, and prognosis as research progresses (15–17). Several studies have confirmed the significant predictive value of an elevated NLR before surgery or chemoradiotherapy in solid tumors, such as gastric cancer (18), lung cancer (19), breast cancer (20), liver cancer (21), pancreatic cancer (22), and kidney cancer (23). Among them, Nakamura et al. (24) showed that high NLR in patients with esophageal cancer predicted poor survival outcomes. Changes in inflammatory response and immune system regulation may improve the survival outcome of esophageal cancer patients. NLR is a simple and low-cost independent prognostic marker (24). The study of Sharaiha et al. (25) also found that NLR reflected the systemic inflammatory response generated by tumors and affected the invasive and metastatic tendency of tumors. Numerous studies have demonstrated the association of NLR with the effectiveness and prognosis of radiotherapy, chemotherapy, immunotherapy, and targeted therapy for esophageal cancer (26–28).

Although numerous studies reported the value of NLR in predicting esophageal cancer patients’ prognosis (29–35), there is no conclusive evidence to confirm whether preoperative NLRs are significantly associated with the long-term prognosis of esophageal cancer patients underwent esophagectomy due to differences in the optimal cutoff, esophageal cancer type, detection timing, and other factors between studies. Therefore, the study aimed at exploring the potential association of preoperative NLR with the long-term prognosis after radical surgery for esophageal cancer through systematic literature search and meta-analysis and to provide evidence-based medical evidence for the construction of accurate prediction models for patients with esophageal cancer.

## Methods

### Literature search

This meta-analysis was performed according to the PRISMA (Preferred Reporting Items for Systematic Reviews and Meta-Analysis) 2020 statement (36) and has been prospectively registered in the PROSPERO (CRD42024524824). We conducted a systematic literature search via PubMed, Embase, Web of Science, and Cochrane up to January 2024 for studies that evaluated the prognostic value of preoperative NLR for the survival outcomes of patients with esophageal cancer. We searched the literature through the following terms: “esophageal neoplasms,” “lymphocytes,” and “neutrophil.” The detailed search strategies are as follows: ((((“Neutrophils”[Mesh]) OR ((((((((((Neutrophil) OR (Polymorphonuclear Neutrophils)) OR (Neutrophil, Polymorphonuclear)) OR (Polymorphonuclear Neutrophil)) OR (LE Cells)) OR (Cell, LE)) OR (LE Cell)) OR (Neutrophil Band Cells)) OR (Band Cell, Neutrophil)) OR (Neutrophil Band Cell))) AND ((“Lymphocytes”[Mesh]) OR (((((Lymphocyte) OR (Lymphoid Cells)) OR (Cell, Lymphoid)) OR (Cells, Lymphoid)) OR (Lymphoid Cell)))) AND (ratio)) AND ((“Esophageal Neoplasms”[Mesh]) OR (((((((((Esophageal Neoplasm) OR (Esophagus Neoplasm)) OR (Esophagus Neoplasms)) OR (Cancer of Esophagus)) OR (Cancer of the Esophagus)) OR (Esophagus Cancer)) OR (Esophagus Cancers)) OR (Esophageal Cancer)) OR (Esophageal Cancers))). Furthermore, we manually screened the bibliography lists of all included studies. Two authors (X.W. and S.J.L.) retrieved and assessed eligible articles independently. Any differences in literature retrieval were resolved by discussion.

### Inclusion and exclusion criteria

Articles were eligible when meeting the following standards: (1) study design was randomized controlled trial, cohort, or case control; (2) studies were performed in patients with esophageal cancer; (3) studies evaluated the prognostic value of preoperative NLR for the survival outcomes of patients with esophageal cancer; (4) at least one survival outcome [overall survival (OS), disease-free survival (DFS), relapse-free survival (RFS), progression-free survival (PFS), cancer-specific survival (CSS), etc.] was evaluated; (5) complete data to analyze risk ratio (RR), odds ratio (OR), or hazard ratio (HR). We excluded study protocols, unpublished studies, non-original studies (including letters, comments, abstracts, correction, and reply), studies without sufficient data, and reviews.

### Data abstraction

Data abstraction was conducted by two authors (X.W. and S.J.L.) severally. Any differences were settled by another author (F.W.L.). The following information extracted from eligible studies: first author name, published year, study duration, study country, study design, research population, sample size, age, gender, TNM stage, NLR cutoff, HR for OS, DFS, RFS, and CSS. If the research data were insufficient, corresponding authors were contacted to request complete data, if available.

### Quality evaluation

The Newcastle-Ottawa Scale (NOS) was applied for assessing the quality of included cohort studies (37), and studies with 7–9 points were considered as high quality (38). Studies with NOS scores below 7 were not included for quantitative analysis. Two authors (X.W. and S.J.L.) severally assessed the quality of all included studies, and any disagreement was settled by discussion.

### Statistical analysis

Meta-analysis was conducted by using Review Manager 5.4.1. HR was used for the synthesis of survival data. Metrics were presented with 95% confidential intervals (CIs). The chi-squared (χ^2^) test (Cochran’s *Q*) and inconsistency index (*I* ^2^) were applied for the evaluation of the heterogeneity (39). χ^2^ *p <* 0.1 or *I* ^2^ more than 50% were regarded as high heterogeneity. Random-effects model was applied to calculate the total HR for outcomes with significant heterogeneity (χ^2^ *p <* 0.1 or *I* ^2^ more than 50%). Or else, fixed-effects model used. In addition, subgroup analyses performed for outcomes with five or more studies included to evaluate the possible confounders, if data were sufficient. In addition, sensitivity analysis was conducted to assess the influence of every included study on the total HR for results with three or more studies included. Egger’s regression tests and funnel plots (40) performed by Review Manager 5.4.1 and Stata 15.1 (Stata Corp, College Station, Texas, USA) to evaluate the publication bias. *p* < 0.05 represents statistically significant publication bias.

## Results

### Literature retrieval, study characteristics, and baseline

**Figure 1:**
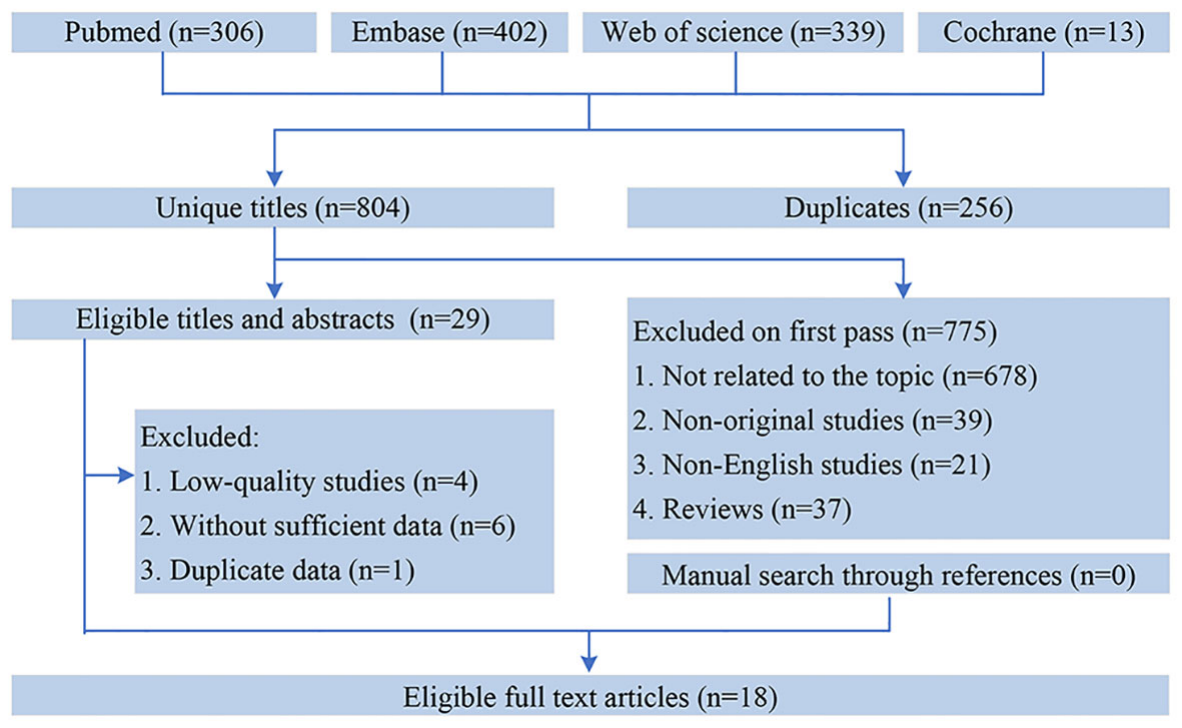
Flowchart of the systematic search and selection process.

**Figure 1** showed the flowchart of the literature retrieval and selection process. A total of 1,060 related studies in PubMed (*n* = 306), Embase (*n* = 402), Web of Science (*n* = 339), and Cochrane (*n* = 13) were identified. A total of 804 literature retained after removing duplicate studies. Eventually, 18 retrospective cohort studies with 6,119 patients were included (24, 25, 29–35, 41–49). **Table 1** presents the characteristics and quality evaluation of each eligible cohort study.

**Table 1:** Characteristics of eligible studies and assessment of risk of bias.

Authors	Study duration	Country	Study design	Population	No. of patients	Gender (male/female)	Mena/median age (years)	TNM stage	NLR cutoff	Confounder adjustment	Quality score
Xu 2022 (29)	2016–2018	China	Retrospective cohort	Esophageal squamous cell carcinoma	370	245/125	61	I–IV	1.304	NA	8
Duan 2015 (14)	2000–2007	China	Retrospective cohort	Patients with histologically confirmed primary ESCC and who were treated with curative-intent esophagectomy	371	276/95	57	I–III	3	Tumor location, differentiation, pT status, pN status	8
Hirahara 2017 (46)	2006–2014	Japan	Retrospective cohort	Patients who underwent potentially curative esophagectomy with R0 resection for histologically verified esophageal squamous cell carcinoma	147	132/15	65.8	I–III	1.6	NA	8
Xiao 2016 (49)	2007–2014	China	Retrospective cohort	Patients with pathologically diagnosed BSCCE who underwent a curative esophagectomy	121	106/15	62	I–III	1.77	NA	7
Han 2019 (43)	2011–2015	China	Retrospective cohort	Esophageal cancer undergoing potentially curative esophagectomy	108	77/31	NA	I–IV	1.88	NA	9
Chen 2020 (35)	2006–2012	China	Retrospective cohort	Patients who had historically proven ESCC and underwent R0 resection	178	139/39	56	I–III	1.89	Tumor length, tumor stage, PLR, LMR	8
Kato 2021 (30)	2010–2015	Japan	Retrospective cohort	ESCC patients who underwent minimally invasive esophagectomy	174	154/20	NA	T1–T4	1.9	NA	7
Shang 2020 (33)	2005–2015	China	Retrospective cohort	Patients with esophageal cancer who had accepted the radical esophagectomy	1883	1550/333	60	I–IV	2.06	NA	7
Fujiwara 2021 (31)	2010–2019	Japan	Retrospective cohort	Patients with biopsy-proven invasive esophageal SCC and no history of previous treatment, who underwent esophagectomies with two- or three-field lymphadenectomies,	111	93/18	66.3	I–IV	2.272	Age, sex, cancer site, pTNM stage, lymph node metastasis, operative procedure, tumor length, and depth of tumor invasion	7
Nakamura 2017 (24)	2005–2016	Japan	Retrospective cohort	Patients with T1 esophageal cancer who underwent subtotal esophagectomy	245	219/26	NA	T1	2.42	NA	7
Hu 2020 (34)	2010–2012	China	Retrospective cohort	Patients with esophageal cancer (EC) underwent radical resection in the cancer center	556	420/136	59	I–IV	2.43	NA	7
Han 2015 (44)	2007–2008	China	Retrospective cohort	Patients underwent radical surgery for pathologically proven ESCC	218	177/41	60.5	I–III	2.6	Tumor diameter, lymph node metastasis	8
Ikeguchi 2016 (47)	2007–2013	Japan	Retrospective cohort	Patients with clinical stages I–III thoracic ESCCs underwent thoracic esophagectomy plus three−field lymph node dissection (cervical, thoracic and abdominal)	84	73/11	65.7	I–III	3	Tumor stage, prognostic nutritional index	7
Ishibashi 2018 (48)	2009–2014	Japan	Retrospective cohort	Patients underwent transthoracic esophagectomy for esophageal cancer	143	121/22	NA	I–IV	3	NA	8
Zhang 2020 (32)	2009–2014	China	Retrospective cohort	Patients with esophageal cancer, who underwent radical surgery (the primary lesions of esophageal cancer were completely resection and postoperative pathology showed negative resection margins, and lymph nodes around the esophagus cancer were dissected)	271	221/50	61	I–IV	3.14	NA	9
He 2017 (45)	2000–2010	China	Retrospective cohort	Patients diagnosed as ESCC and accepted radical resection	317	268/49	60	I–IV	3.3	Gender, lymphatic metastasis, tumor size, tumor size, tumor differentiation, PLR	8
Feng 2014 (42)	2005–2008	China	Retrospective cohort	Patients with ESCC who underwent curative esophagectomy	483	411/72	59.1	T1–T4	3.5	NA	7
Sharaiha 2011 (25)	1996–2009	USA	Retrospective cohort	Patients with histologically confirmed esophageal cancer who had undergone esophagectomy	339	237/102	62.8	I–IV	5	Neutrophils, lymphocytes, age, sex, race, stage, histology, tumor differentiation	8

### OS

**Figure 2:**
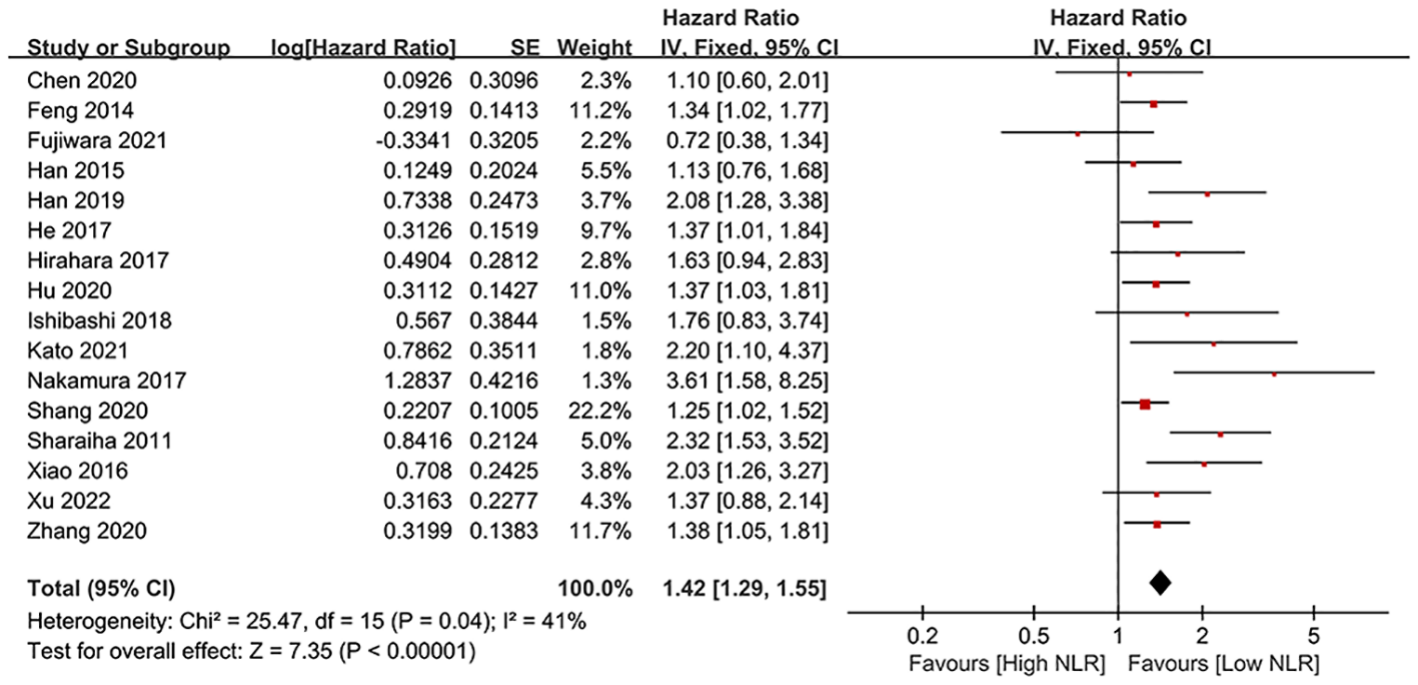
Forest plots of OS.

Results of OS were synthesized from 16 cohort studies (24, 25, 29–35, 42–46, 48, 49), and meta-analysis revealed a significantly shorter OS in high NLR group (HR: 1.47; 95% CI: 1.29, 1.67; *P* < 0.00001). There was no significant heterogeneity (*I* ^2^ = 41%, *P* = 0.04) (**Figure 2**). Subgroup analysis based on mean/median age, NLR cutoff and region revealed that difference remained significant in all subgroups (**Table 2**).

**Table 2:** Subgroup analysis of OS and DFS.

Subgroup	OS				DFS			
	Study	HR [95% CI]	*P*-value	*I* ^2^	Study	HR [95% CI]	*P*-value	*I* ^2^
Total	16	1.42 [1.29–1.55]	< 0.00001	41%	8	1.62 [1.29–2.05]	< 0.0001	57%
Mean/median age								
> 60 years	7	1.46 [1.14–1.86]	0.003	56%	3	1.79 [1.12–2.85]	0.02	60%
≤ 60 years	5	1.30 [1.15–1.47]	< 0.0001	0%	3	1.28 [1.06–1.54]	0.01	0%
NLR cutoff								
≥ 3	5	1.47 [1.27–1.71]	< 0.00001	30%	3	1.78 [1.14–2.80]	0.01	64%
< 3	11	1.38 [1.23–1.56]	< 0.00001	48%	5	1.57 [1.14–2.16]	0.006	62%
Region								
China	10	1.36 [1.231.50]	< 0.00001	0%	5	1.37 [1.11–1.69]	0.003	37%
Japan	5	1.69 [1.02–2.79]	0.005	63%	2	2.63 [1.62–4.27]	< 0.0001	0%
USA	1	2.32 [1.53–3.52]	< 0.0001	/	1	2.26 [1.44–3.55]	0.0004	/

### DFS

**Figure 3:**
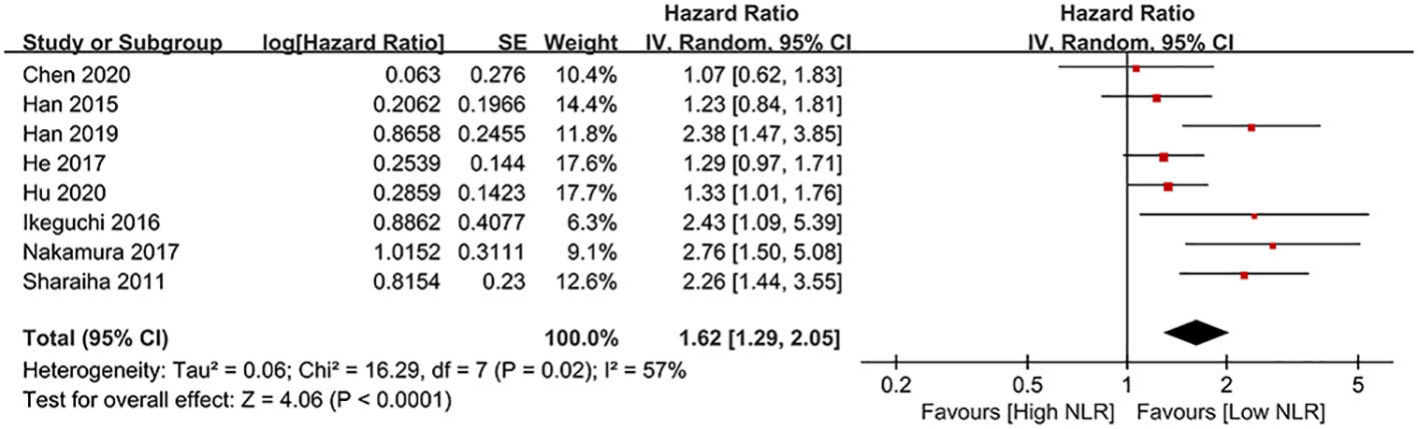
Forest plots of DFS.

Results of DFS were synthesized from eight cohort studies (24, 25, 34, 35, 43–45, 47), and exhibited a significantly shorter DFS in high NLR group (HR: 1.62; 95% CI: 1.29, 2.05; *P* < 0.0001). Significant heterogeneity exists (*I* ^2^ = 57%, *P* = 0.02) (**Figure 3**). Subgroup analysis based on mean/median age, NLR cutoff and region showed significant difference in all subgroups (**Table 2**).

### RFS

**Figure 4:**
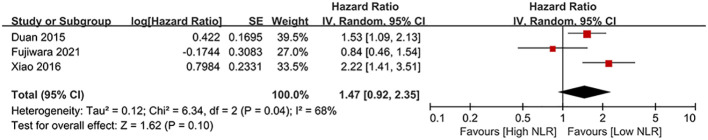
Forest plots of RFS.

Results of RFS were synthesized from three cohort studies (31, 41, 49), and meta-analysis revealed similar RFS exists in all groups (HR: 1.47; 95% CI: 0.92, 2.35; *P* = 0.10). Significant heterogeneity shown (*I* ^2^ = 68%, *P* = 0.04) (**Figure 4**).

### CSS

**Figure 5:**
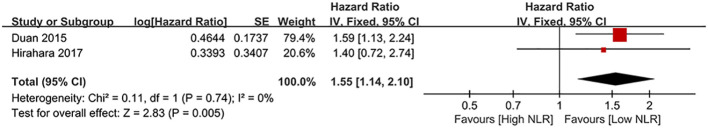
Forest plots of CSS.

Results of CSS were synthesized from two cohort studies (41, 46), and meta-analysis found a significantly shorter CSS in high NLR group (HR: 1.62; 95% CI: 1.29, 2.05; *P* < 0.0001). No significant heterogeneity (*I* ^2^ = 0%, *P* = 0.74) (**Figure 5**).

Publication bias and sensitivity analysis

**Figure 6:**
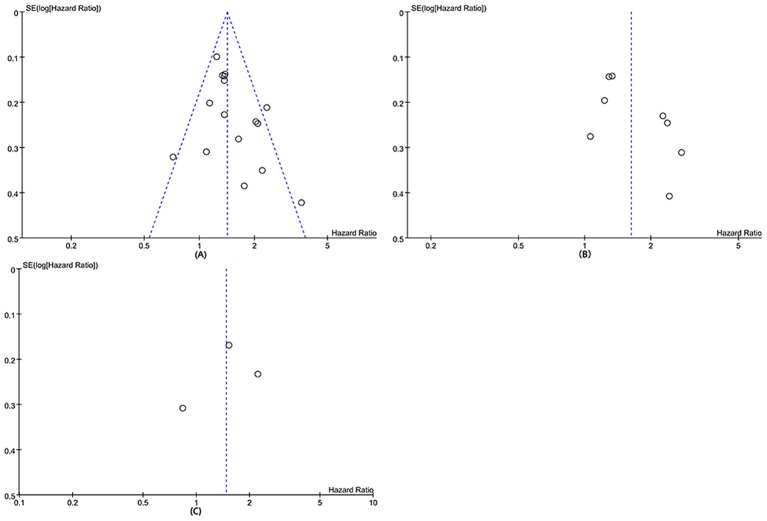
Funnel plots of OS **(A)**, DFS **(B)**, and RFS **(C)**.

The potential publication bias of OS, DFS, RFS were assessed through funnel plots and Egger’s regression tests. No publication bias existed in OS (Egger’s test *P* = 0.098) (**Figure 6A**), DFS (Egger’s test *P* = 0.093) (**Figure 6B**), and RFS (Egger’s test *P* = 0.708) (**Figure 6C**). In addition, sensitivity analysis was used to assess the effect of each cohort study on the total HR. By excluding eligible studies one by one, the sensitivity analysis of OS, DFS, and RFS was performed.

**Figure 7:**
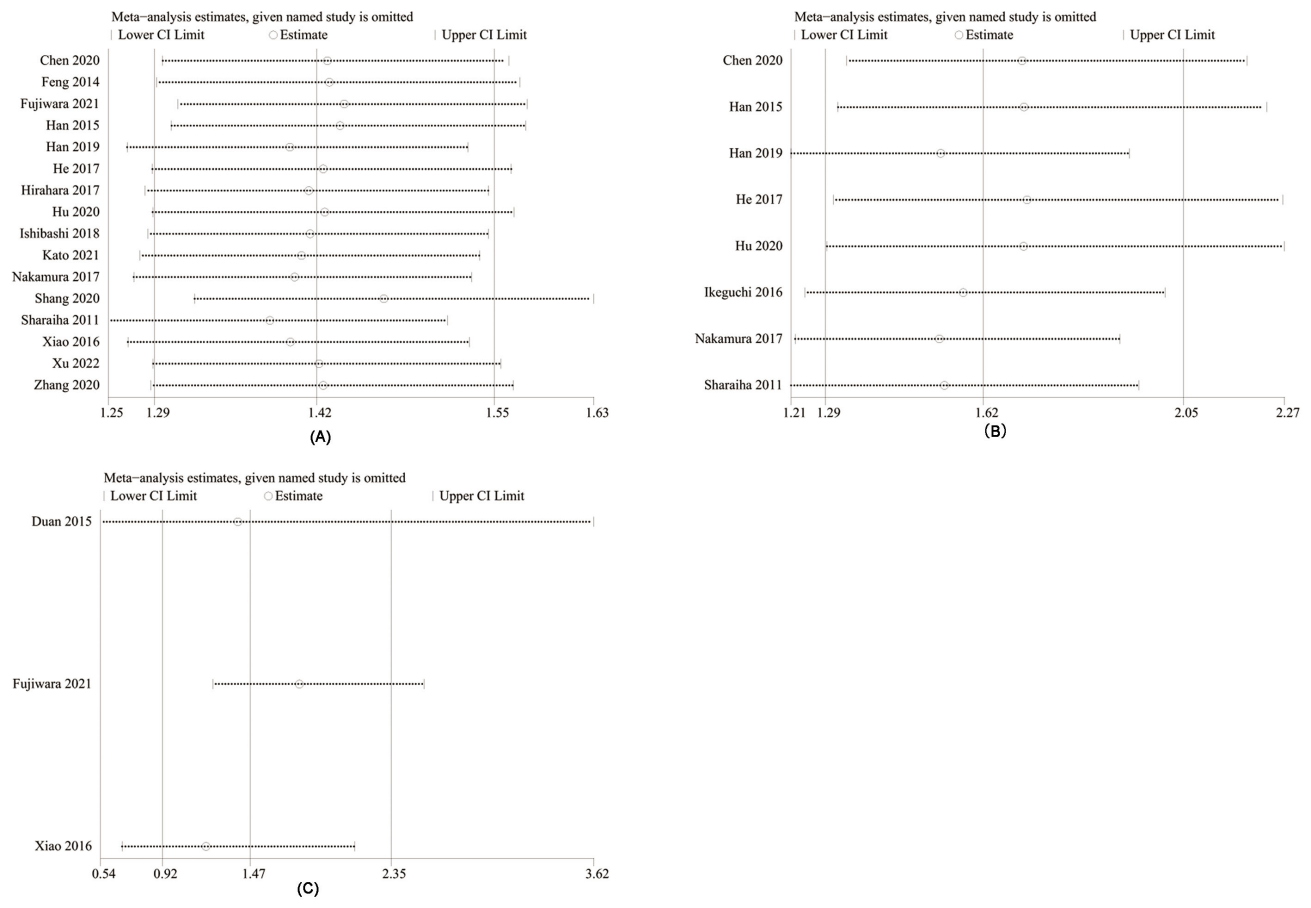
Sensitivity analysis of OS **(A)**, DFS **(B)**, and RFS **(C)**.

Sensitivity analysis exhibited that total HR kept stable after removing of each cohort study for OS (**Figure 7A**), DFS (**Figure 7B**), and RFS (**Figure 7C**), which implied the low sensitivity and stability of results.

## Discussion

At present, the comprehensive treatment method mainly based on the radical resection, which is generally applied for patients occurred esophageal cancer (50). Several studies reported notable survival rate and recurrence disparities among esophageal cancer patients across different stages, with stages I and II patients displaying significantly superior 5-year postoperative survival rates compared to stages III and IV patients (51, 52). Therefore, the selection of effective indicators for preoperative clinical staging diagnosis, postoperative recurrence, and metastasis assessment of patients with esophageal cancer is conducive to formulating the best treatment for patients and intervention programs for high-risk groups of recurrence and metastasis. More attention focused on the relationship between NLR and tumor occurrence, development, prognosis, with the deepening researches on tumor and tumor microenvironment. Studies have proved that the higher level of preoperative NLR predicts, the worse prognosis (53–56). Dong et al. (57) found that the OS in patients with high-preoperative NLR was significantly shorter than the low, and CSS in patients with high NLR was also significantly shortened. Multivariate Cox models implied that preoperative NLR was affected by OS and CSS, after adjusting the clinical and pathological features. Meanwhile, clinical and pathological characteristics have no effect on the prognosis of esophageal cancer patients (43). Therefore, NLR can be used as an independent prognostic factor in esophageal cancer patients.

Here, several survival outcomes analyzed for assessing the value of preoperative NLR among patients occurred esophageal cancer. Our results revealed a significantly shorter OS, DFS, and CSS in high NLR group. In addition, subgroup analysis of OS and DFS based on mean/median age, NLR cutoff and region reveled significant difference exists in all subgroups, suggesting that preoperative NLR was significantly associated with the prognosis of esophageal cancer patients underwent esophagectomy, more attention should be paid to the clinical treatment of esophageal cancer. Our findings support most of the previously published research (57–59). It is worth mentioning that whether tumor stage affects the prognostic value of NLR for esophageal cancer is still controversial. The Global Esophageal Cancer Collaboration provided data on 22,654 patients with epithelial esophageal cancer. Of these, 13,300 patients who had not been treated before surgery were pathologically evaluated after esophagectomy or endoscopic therapy. Risk-adjusted survival was calculated for each patient using randomized survival forest analysis to identify relevant pathological stage groups, where survival decreased as the group increased, with significant differences between groups and homogeneity within groups. Although pTis had similar survival rates to stage pT1N0M0 cancer, it was placed in stage 0 alone. Thus, the final 0–IV stage is obtained with different stages and different prognoses (60). Studies have shown that preoperative NLR is significantly correlated with preoperative treatment and T stage (*P* = 0.0005 and *P* < 0.0001, respectively), but preoperative NLR is not significantly correlated with tumor stage (*P* = 0.16) or other evaluation characteristics (61, 62). In addition, a number of studies have shown that preoperative NLR level has no significant correlation with T stage and N stage, and the preoperative NLR level is not affected by T stage and N stage (31, 35, 43). In addition, another study showed that the impact of prognostic outcomes in patients with esophageal cancer does not change based on clinical and pathological features (43). In conclusion, most of the existing evidence supports that NLR can be used as an independent prognostic factor for patients with esophageal cancer, and its predictive value is not affected by confounding factors such as tumor stage. The cost of obtaining this parameter is relatively low; it is easy to evaluate in most medical centers and as an objective indicator, the bias caused by NLR is relatively limited between laboratories.

Lymphocytes play a vital role in inhibiting the proliferation, metastasis, cytotoxicity, and cell death of tumor cells, while neutrophils can produce cell growth factors, chemokines, and proteases, which can promote the growth of tumor cells. It can promote the proliferation and differentiation of tumor cells by remodeling extracellular matrix framework and inhibiting immune function (63, 64). Therefore, NLR is a balance index reflecting the anti-tumor immunity, tumor cell growth, and inflammation (65, 66). The tumors invasion into lymph nodes can result in the loss of immune function, less lymphocytes, and higher NLR value (29). This study showed that the OS, DFS, and CSS of the high NLR group were significantly shorter than the low. Studies have shown that NLR affects the prognosis of esophageal cancer patients significantly because tumor-associated neutrophils contain and secrete factors. These factors, such as human leukocyte elastase, peroxidase, matrix metalloproteinase, heparin-binding growth factor, and vascular permeability factor can promote angiogenesis and inhibit cell adsorption (65). Therefore, the increase of tumor-associated neutrophils was positively correlated with tumor progression. Increased tumor-associated neutrophils can inhibit the anticancer activity of lymphocytes, natural killer cells, and activated T cells (3, 67). Finally, progressive decline in nutritional status, biological function, and other adverse events resulted from systemic inflammatory responses among patients with cancer (68). Therefore, high NLR may have an indirect adverse effect on tumor patients, resulting in adverse patient outcomes.

To some extent, this study has revealed the significance of NLR, a commonly used hematological index in clinical practice, for the prognosis of esophageal cancer patients, but there are still some limitations. First, there was no standard method of electing and calculating the NLR best truncation value currently, and the common methods are ROC curve and median. Due to the different calculation methods of the NLR truncation values in the literatures included, the difference between the number of cases and the NLR truncation values were large, resulting in a certain degree of heterogeneity in the results. Second, although the subjects included were all patients with esophageal cancer, the basic characteristics, clinical stage, pathological type, degree of tumor invasion, and postoperative treatment of patients with esophageal cancer in each literature were different. Due to the relatively small sample size, all confounding factors could not be taken into account in the subgroup analysis. Moreover, the studies were all retrospective studies with a relatively small sample size, and most of them are single-center data from Asian countries (mainly China and Japan), which leads to a certain degree of unavoidable bias.

Under the situation, this meta-analysis is the latest and largest study to report the prognostic value of preoperative NLR for the survival outcomes of esophageal cancer patients underwent esophagectomy. The findings of this study support the importance of paying attention to the level changes of NLR in the clinical treatment of esophageal cancer patients underwent esophagectomy and building a more valuable prediction model based on inflammatory indicators including NLR to improve the prognosis and life quality of esophageal cancer patients.

## Conclusion

Meta-analysis demonstrated that preoperative NLR can be used as an independent prognostic factor among esophageal cancer patients underwent esophagectomy. High-preoperative NLR is associated with poor prognosis in patients with esophageal cancer. Considering the limitations of retrospective studies, potential selection bias, and unavoidable heterogeneity, more large-scale, multicenter prospective clinical studies are needed to further validate the relationship between preoperative NLR and prognosis of esophageal cancer.
